# Malaria incidence among children less than 5 years during and after cessation of indoor residual spraying in Northern Uganda

**DOI:** 10.1186/s12936-017-1966-x

**Published:** 2017-08-07

**Authors:** Allen E. Okullo, Joseph K. B. Matovu, Alex R. Ario, Jimmy Opigo, Humphrey Wanzira, David W. Oguttu, Joan N. Kalyango

**Affiliations:** 1 Uganda Public Health Fellowship Programme, Ministry of Health, P.O. Box 7272, Kampala, Uganda; 2 Ministry of Health, Kampala, Uganda; 3 University of Trieste, Piazzale Europa, Trieste, Italy; 4 Makerere University College of Health Sciences, Kampala, Uganda

**Keywords:** Malaria incidence, Malaria epidemic, Indoor residual spraying

## Abstract

**Background:**

In June 2015, a malaria epidemic was confirmed in ten districts of Northern Uganda; after cessation of indoor residual spraying (IRS). Epidemic was defined as an increase in incidence per month beyond one standard deviation above mean incidence of previous 5 years. Trends in malaria incidence among children-under-5-years were analysed so as to describe the extent of change in incidence prior to and after cessation of IRS.

**Methods:**

Secondary data on out-patient malaria case numbers for children-under-5-years July 2012 to June 2015 was electronically extracted from the district health management information software2 (DHIS2) for ten districts that had IRS and ten control districts that didn’t have IRS. Data was adjusted by reporting rates, cleaned by smoothing and interpolation and incidence of malaria per 1000 population derived. Population data obtained from 2002 and 2014 census reports. Data on interventions obtained from malaria programme reports, rainfall data obtained from Uganda National Meteorological Authority. Three groups of districts were created; two based on when IRS ended, the third not having IRS. Line graphs were plotted showing malaria incidence vis-à-vis implementation of IRS, mass net distribution and rainfall. Changes in incidence after withdrawal of IRS were obtained using incidence rate ratios (IRR). IRR was calculated as incidence for each month after the last IRS divided by incidence of the IRS month. Poisson regression was used to test statistical significance.

**Results:**

Incidence of malaria declined between spray activities in districts that had IRS. Decline in IRR for 4 months after last IRS month was greater in the sprayed than control districts. On the seventh month following cessation of IRS, incidence in sprayed districts rose above that of the last spray month [1.74: 95% CI (1.40–2.15); and 1.26: 95% CI (1.05–1.51)]. Rise in IRR continued from 1.26 to 2.62 (95% CI 2.21–3.12) in June 2015 for districts that ended IRS in April 2014. Peak in rainfall occurred in May 2015.

**Conclusion:**

There was sustained control of malaria incidence during IRS implementation. Following withdrawal and peak in rainfall, incidence rose to epidemic proportions. This suggests a plausible link between the malaria epidemic, peak in rainfall and cessation of IRS.

## Background

Malaria is still a major public health challenge in most of the sub-Saharan African countries worldwide. In 2015, of the estimated 214 million cases of malaria, 88% were diagnosed in this region alone [1] with the highest burden shared by pregnant women and children under 5 years.

Malaria is endemic in over 95% of Uganda [2]. Over the last decade, the National Malaria Control Programme with the support of donors and implementing partners adopted a number of effective strategies [3–12] to reduce this disease burden [2, 13]. These included provision and use of long-lasting insecticide-treated bed nets (LLINs), diagnosis and prompt treatment using World Health Organization (WHO) recommended anti-malarial medicines, and intermittent preventive therapy (IPT) for pregnant women. In addition to this, indoor residual spraying (IRS) using bendiocarb, a carbamate was implemented in ten districts of Northern Uganda that had the highest burden, from 2010 to 2014. As a result, a marked decline was observed in the mid-North where the prevalence of malaria reduced from 63% in 2009 to 20% in 2014 with the districts in which IRS had been implemented having the lowest prevalence of 7% [14]. This decline in burden was followed by the withdrawal of IRS in 2014.

Evidence confirms that malaria control using IRS can result in overall malaria transmission reduction or even elimination and has been associated with a 62% reduction in malaria incidence when applied [11]. For this reason, it is not surprising that the northern districts in which IRS was implemented had the greatest decline in malaria. IRS is effective for 3–6 months with carbamate spray chemicals depending on the type of surface on which it is sprayed [3].

**Fig. 1:**
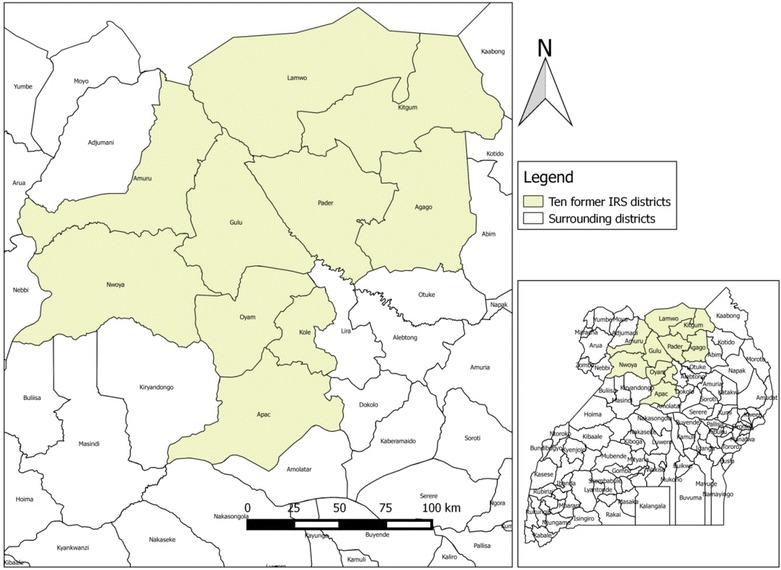
Location of the ten former IRS districts under study

There was a turn of events in May 2015 when an upsurge in malaria cases started during the peak in rainfall in all ten northern districts where IRS had been implemented as reported through electronic health management information systems (eHMIS) in the district health information software 2 (DHIS2). Most cases were among children below 5 years of age in the ten districts of Amuru, Apac, Kitgum, Nwoya, Lamwo, Agago, Gulu, Kole, Oyam and Pader (see Fig. 1 for details). A malaria epidemic was declared in June 2015. Epidemic in this study refers to an increase in incidence per month beyond one standard deviation above the mean incidence of the previous 5 years. The values used for the epidemic threshold differed by month for each district based on individual district means and standard deviations for the previous 5 years. The incidence values, per 1000 population, used for the epidemic threshold of all ten Northern Uganda districts grouped by month from January to December were: 55, 44, 47, 56, 70, 74, 45, 43, 41, 48, 44 & 32.

The Malaria Indicator Survey report for 2014 [14] revealed that there was 67% coverage of LLINs in the mid-north region, and usage of 79% in children-under-5-years. This translated into a low LLIN usage of 53% in the face of IRS withdrawal according to the WHO assessment of the malaria epidemic in 2015. In addition, the WHO assessment of the malaria epidemic in 2015 reported that after cessation of IRS there was no adequate surveillance system in place to detect the increase and initiate an appropriate outbreak response.

Evidence shows that increases in malaria cases might highlight the fragility of malaria control and the need to maintain control programmes even if numbers of cases have been reduced substantially [15]. Sustaining gains made in malaria control programmes has been a challenge where technical problems exist such as lack of resources for annual renewal of each intervention [8]. Strengthening other interventions, such as malaria case management, provision and optimal use of LLINs, malaria surveillance and behavioural change communication has been recommended by the WHO as a way of sustaining gains made by IRS during its exit.

This study describes the trends in incidence of malaria among children under-5 years of age and compares IRRs during and after withdrawal of IRS for the period July 2012 to June 2015. The aim is to assess the change in the burden of malaria among the under-5-year-olds prior to and following withdrawal of IRS.

## Methods

### Study design

A secondary data analysis was conducted using health facility data via the eHMIS, population data from 2002 and 2014 census reports; IRS implementation data from project reports; and data on LLIN coverage and use from the malaria indicator survey report. Data on passive malaria surveillance and behavioural change communication (BCC) was collected from malaria programme reports and district malaria focal persons. Data on rainfall was collected from the Uganda National Meteorological Authority.

Malaria incidence data and incidence rate ratio (IRR) were calculated comparing malaria incidence prior to and after cessation of implementation of IRS. Different data included in this analysis were: malaria case data for under-5-year-old malaria cases obtained from eHMIS; population data from census reports; programme data on IRS implementation; LLIN universal campaign distribution data; data on LLIN coverage and use among under-5-year-olds; and data on surveillance and BCC post IRS within the 10 epidemic districts over the period July 2012 and June 2015.

### Description of districts under study

The analysis used malaria data from ten epidemic districts of Northern Uganda namely: Lamwo, Kitgum, Gulu, Nwoya, Amuru, Pader, Agago, Apac, Oyam and Kole, as shown in Fig. 1. There were 10 surrounding control districts, namely: Alebtong, Abim, Amolatar, Adjumani, Arua, Kaabong, Lira, Nebbi, Otuke and Kotido. These districts have similar climatic conditions and rainfall seasonality patterns with temperatures ranging between 16 and 32 °C, relative humidity of 50–80% and two rainy seasons, from April to May and August to September, annually. These conditions are suitable for and promote malaria transmission, the peak seasons of which occur just after the two rainy seasons. At the start of the epidemic, Lamwo had an estimated population of 134,379; Agago, 227,792; Kitgum, 204,048; Nwoya, 133,506; Amuru, 186,696; Gulu, 436,345; Pader, 178,004; Kole, 239,327; Oyam, 383,644; and Apac, 368,626 based on the results for the 2014 national population and housing census [16].

### Description of the HMIS dataset in DHIS2

Data on malaria cases for this study was obtained from the eHMIS, in DHIS2, for Uganda. In January 2011, Uganda adopted the electronic HMIS. Full scale implementation of DHIS2 begun in January 2012 which involved piloting it in four districts in the western region [17]. National roll out of DHIS2 took place in 2012 and involved the training of all district health officers, district biostatisticians, HMIS focal persons at district level and health sub districts as well as surveillance officers on the electronic HMIS (DHIS2) [17]. Prior to this, Uganda used the paper based system in which district data collected as aggregates were submitted to the Ministry of Health resource center, currently the Division of Health Information, and entered in databases such as EpiInfo, web-enabled databases and Microsoft Excel stored in the data bank.

The use of this data reporting system was not consistent between 2012 and 2015 with initially low monthly reporting rates within each of the districts in 2012. This has progressively improved over the years as a result of HMIS assessments and trainings as well as support supervision of health facilities. To control for the monthly variation in reporting within each district, specific monthly reporting rates were used to adjust total malaria cases. Also, smoothing and interpolation methods were used to fill in missing figures between months in the earlier years. The use of RDTs along with training on integrated management of malaria (IMM) was rolled out in 2011/12 which was prior to the study period. Consequently, the testing rates increased overtime from 30% laboratory confirmed malaria cases between July 2012 and June 2013, to 49% confirmed between July 2013 and June 2014 to 86% confirmed malaria cases between July 2013 and June 2015.

Both public and private health facilities from all districts report data on malaria cases to this system on a weekly, monthly and quarterly basis using specific reporting tools. Data on malaria cases in all districts is captured on a daily basis at the facility level using daily registers. At the community level, data collected by village health teams (VHTs)—a category of community health workers—are aggregated with facility-level data before they are reported into DHIS2 on a weekly basis. These data are aggregated and reported into DHIS2 each month. To avoid double counting malaria inpatient cases, which are initially registered at the outpatient department, the HMIS 105, an Outpatient monthly reporting tool, was used to access data on malaria cases. Total malaria cases for 0–4 years was the indicator used in HMIS 105. This indicator includes both laboratory and clinically diagnosed cases of malaria. Prior to June 2015, data on malaria cases was not disaggregated based on diagnosis. Data on reporting rates for HMIS 105 reports for each month in each district for the period July 2012 to June 2015 was also obtained in DHIS2.

### Description of the population data

The monthly populations per district were based on monthly projections from mid-year population estimates for each district for-2012, 2013 and 2014 populations obtained from the National Population and Housing census reports of 2002 and 2014. The population of those under-5 years of age in each district per month, was 0.17% of the overall projected populations derived from National Population and Housing Census reports.

### Description of data on malaria interventions

The interventions assessed included IRS, universal coverage campaign with LLINs, LLIN coverage and net use among under-5-year-olds following the universal coverage campaign. Obtaining information on the interventions took place between October 2015 and November 2016. Information on the malaria control interventions implemented during the period July 2012 to June 2015 was obtained from programme reports and through communication with the malaria control officers at the Ministry of Health and at the respective districts.

### Description of the meteorological dataset

Data on total rainfall received in millimeters per month for the period July 2012 to June 2015 was included in this study. Averages per month of the total rainfall received from each of three districts in Northern Uganda, (Gulu, Kitgum and Lira) were selected as being representative of the ten districts being analysed. Also, the weather stations in the other districts were largely non-functional during this period. Data on rainfall is collected daily using rain gauges at functional weather stations managed by the Uganda National Meteorological Authority. Daily rainfall received is then summed up to get monthly totals. This is then reported and shared in excel spreadsheets.

### Study variables

The dependent variable in this analysis was the incidence of malaria among children under-5-years in the ten former IRS districts for each of the months for the period July 2012 to June 2015. The main independent variable was application of IRS. Other variables included in the analysis were: rainfall, reporting rates for of reporting malaria cases in the districts of interest and coverage of LLINs in the mass distribution.

### Data analysis

Data on total malaria cases and reporting rates obtained from DHIS2 were exported to MS Excel. The number of total malaria cases was then adjusted using reporting rates per month. Adjustment was done by dividing the total number of malaria cases each month by respective reporting rates to get the estimated number of malaria cases that would have been reported if all (100%) facilities registered on DHIS2 had reported. This was followed by smoothing and interpolation methods to fill in missing figures between months. Data for missing months were interpolated from the last and first available month of the figures adjusted for completeness if only one of two consecutive months were missing. If more than two consecutive months were missing, the relative weight of each month’s results was calculated from the total of all the other years and this weight was then applied to the mean monthly result using the last and first available data point. This provided an estimate of the likely actual number of cases.

DHIS2 malaria case data for those under-5 years of age and population data for under-5 years of age were used to calculate the incidence of malaria among those under-5 years. Malaria incidence was defined as the total number of new cases of malaria per 1000 population. Monthly incidence per district was defined as the total number of new cases of malaria per 1000 population of that particular age-group in the given month in the district. Incidence per month for the ten districts was defined as the total number of new cases per month per 1000 population for all the ten former IRS districts for that particular age-group. Cases are defined as persons who are reported by the health facility as having diagnostically or clinically confirmed malaria. Two groups of districts were created based on when IRS ended. Group I included districts that ended IRS in November 2014 while group II included districts that ended IRS in April 2014. Incidence rate ratios (IRR) were calculated for all the months following IRS activities. A third group of neighboring districts that had not experienced IRS, that is, Adjumani, Arua, Nebbi, Amolatar, Lira, Alebtong, Otuke, Kotido, Abim and Kaabong was added to the comparison in order to check whether variations in malaria incidences could be attributed to malaria seasonality due to rainfall. The months in this group have been numbered to correspond to those in group II so as to be able to make comparisons of similar months since in this group there was no spraying.

MS Excel was used to produce line graphs showing trends in malaria incidence by month and points at which IRS and universal distribution of LLINs was implemented. Data on malaria incidence were presented alongside data on rainfall inform of line graphs to highlight malaria seasonality that follows rainfall patterns.

Poisson regression was used to test statistical significance of the incidence rate ratio (IRR) for each of the months following IRS activities i.e. during application (which was every 6 months) and every month following spray until after cessation of IRS while controlling for rainfall received. The reference months for the IRR were the months in which IRS was implemented. The numerator for each of the IRR values was the incidence for each month following IRS activities. Thus, the IRR was calculated as the incidence for each month after IRS divided by the incidence of the month when IRS took place for each of the groups. Hence, the IRR values showed the change in incidence following IRS by comparing incidences of the months following IRS with incidences of the months when IRS took place. Results for IRR were presented in tables showing the IRR values numbered 1–7 (for the districts that ended IRS in November 2014) and 1–14 (for the districts that ended IRS in April 2014). Given that IRS was conducted every 6 months, values numbered six onwards represent the period after cessation of IRS. Results of the Poisson regression analysis are presented in tables with confidence intervals.

Incidence rate ratios values for ten surrounding non IRS districts were calculated and compared with the IRR values of the former IRS districts to determine the possible effect of IRS on decline in IRR. The post spray period for group two districts, that is, April 2014 to June 2015 was considered to allow for a longer period of observation that covers two transmission seasons.

## Results

### Study populations characteristics

A total of 1453,418 under-5-year old reported malaria cases from both the former IRS and non IRS surrounding control districts were included in the analysis. Of this, 613,399 (42%) were not confirmed by laboratory diagnosis. For the districts that had IRS, a total of 746,285 under-5 year old reported malaria cases were included in the analysis, of this, 360,885 (48%) were not confirmed by laboratory diagnosis. However, there were changes over time with the percentage of unconfirmed malaria cases reducing from 70% between July 2012 and June 2013, to 51% between July 2013 and June 2014 and finally to 24% between July 2014 and June 2015. Malaria case data for the months August and September 2012 in Pader district and for August 2012 for Kole district was found to be missing. This was filled by smoothing and interpolation methods.

### Trends of malaria incidence before and after withdrawal of IRS

**Fig. 2:**
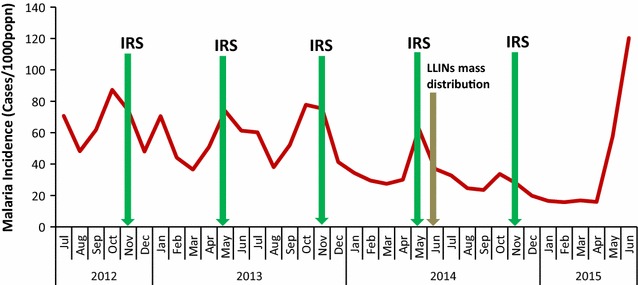
Malaria incidence among under-5 year-olds vis-à-vis control interventions in five districts that ended IRS in November 2014 for period July 2012 to June 2015

**Fig. 3:**
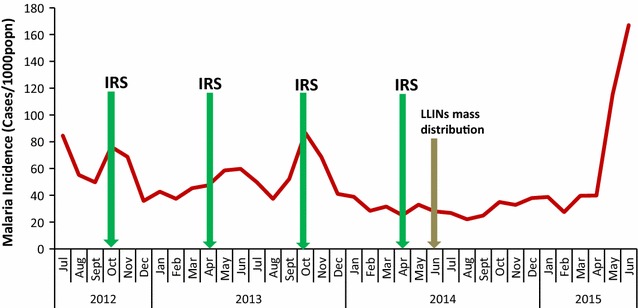
Malaria incidence among under-5 year-olds vis-à-vis control interventions in five districts that ended IRS in April 2014 for period July 2012 to June 2015

Prior to withdrawal of IRS, there was an observed decline in incidence whenever IRS was implemented in group I and II districts, as shown in Figs. 2 and 3 and as evidenced by the IRR for all the months following implementation, Tables 1 and 2. On the contrary, following the withdrawal of IRS, there was an exponential increase in incidence of malaria as evidenced in Figs. 2 and 3, and by IRR values as shown in Tables 1 and 2. As shown in Figs. 1 and 2, large peaks in cases occurred in most cases prior to implementation of IRS.

**Table 1:** Malaria IRR’s in the months following implementation of IRS in group I districts. Source: DHIS2, 2002 & 2014 Census reports

Month	IRR	95% CI
1 (Dec 2014)	0.77	0.64–0.92
2 (Jan 2015)	0.75	0.63–0.90
3 (Feb 2015)	0.49	0.41–0.60
4 (Mar 2015)	0.51	0.42–0.61
5 (Apr 2015)	0.60	0.50–0.72
6 (May 2015)	0.81	0.61–1.07
7 (Jun 2015)	1.74	1.40–2.15

**Table 2:** Malaria IRR’s in the months following implementation of IRS in group II districts. Source: DHIS2, 2002 & 2014 Census reports

Month	IRR	95% CI
1 (May 2014)	0.89	0.78–1.01
2 (Jun 2014)	0.80	0.69–0.91
3 (Jul 2014)	0.70	0.61–0.79
4 (Aug 2014)	0.57	0.49–0.65
5 (Sept 2014)	0.65	0.57–0.73
6 (Oct 2014)	0.71	0.57–0.88
7 (Nov 2014)	1.26	1.05–1.51
8 (Dec 2014)	0.82	0.58–1.15
9 (Jan 2015)	0.87	0.62–1.22
10 (Feb 2015)	0.61	0.41–0.91
11 (Mar 2015)	0.82	0.59–1.14
12 (Apr 2015)	0.61	0.44–0.85
13 (May 2015)	1.77	1.45–2.17
14 (Jun 2015)	2.62	2.20–3.12

Figure 2 shows malaria incidence among under-5 year-olds vis-à-vis control interventions in 5 districts that ended IRS in November 2014 for period July 2012 to June 2015. There was a steady decline in incidence for the first 3 months following implementation of IRS in group one districts. This is evidenced by decreasing IRRs from 0.77 (95% CI 0.64–0.92) to 0.49 (95% CI 0.41–0.60) shown in Table 1. The incidence started to rise from the fourth to the fifth month following IRS during the period prior to withdrawal as shown by increasing IRR’s, that is, 0.51 (95% CI 0.42–0.61) to 0.81 (95% CI 0.61–1.07). However, this increase was still below the baseline incidence during the spray month, which was the comparison month. Following the withdrawal of IRS, the IRR rose from 0.81 (95% CI 0.61–1.07) to 1.74 (95% CI 1.40–2.15) in June 2015 reaching epidemic proportions.

Figure 3 shows malaria incidence among under-5 year-olds vis-à-vis control interventions in five districts that ended IRS in April 2014 for period July 2012 to June 2015. IRS was implemented with an interval of 6 months for the period reviewed and mostly during peak transmission seasons. When IRS was implemented during peak transmission, there was a rapid decline in malaria incidence of up to 53% in the next 2 months following. There was a decline in incidence for the first 4 months following implementation of IRS in group 2 districts. This coincided with the implementation of universal coverage campaign with LLINs in June 2014, after which the incidence of malaria reduced by 28%, from 32 cases per 1000 population in June to 23 cases per 1000 population in August 2014. The reduction in incidence for the first 4 months is further evidenced by decreasing IRR’s from 0.89 (95% CI 0.78–1.01) to 0.57 (95% CI 0.49–0.65), using the incidence for the month when IRS was implemented for comparison as shown in Table 2. IRR started to rise from the fifth month following IRS as shown in Table 2.

Following the withdrawal of IRS from group II districts, the IRR decreased by 0.11 in the month following the spray month. As was the case with group I, IRR rose to epidemic proportions, from 0.71 (95% CI 0.57–0.88) in the sixth month to 2.62 (95% CI 2.20–3.12) in the 14th month (June 2015). For the eighth to the twelfth month after implementation of IRS the apparent reduction in incidence compared to that of the spray month as shown by the IRR values in Table 2 was statistically significant for only February and April 2015. There was a significant rise in malaria incidence in the thirteenth and fourteenth month (May & June 2015) following the cessation of IRS implementation [1.77: 95% CI (1.45–2.17); 2.62: 95% CI (2.21–3.12)].

### Trends of malaria incidence in non IRS districts for period April 2014 to June 2015

In the control districts, the IRR increased by 0.14 in the month following April. This was followed by a decrease in IRR up to the fifth month (September). In October, the sixth after April, the IRR increased to 1.1 following the peak in rainfall season. The IRR then fell below one for the next 6 months following the dry season after which there was a spike in May and June at 1.1 and 1.3 respectively as shown in Table 3.

**Table 3:** Malaria IRR from May 2014 to June 2015 in control districts. Source: DHIS2, 2002 & 2014 Census reports

Month	IRR	95% CI
1 (May 2014)	1.14	1.0–1.30
2 (Jun 2014)	0.97	0.85–1.11
3 (Jul 2014)	0.99	0.88–1.13
4 (Aug 2014)	0.88	0.77–0.99
5 (Sept 2014)	0.95	0.83–1.08
6 (Oct 2014)	1.12	0.91–1.36
7 (Nov 2014)	0.99	0.80–1.21
8 (Dec 2014)	0.77	0.61–0.97
9 (Jan 2015)	0.68	0.53–0.87
10 (Feb 2015)	0.58	0.45–0.75
11 (Mar 2015)	0.58	0.45–0.75
12 (Apr 2015)	0.57	0.44–0.74
13 (May 2015)	1.11	0.91–1.36
14 (Jun 2015)	1.27	1.05–1.54

### Trends of malaria in former IRS districts following rainfall patterns

**Fig. 4:**
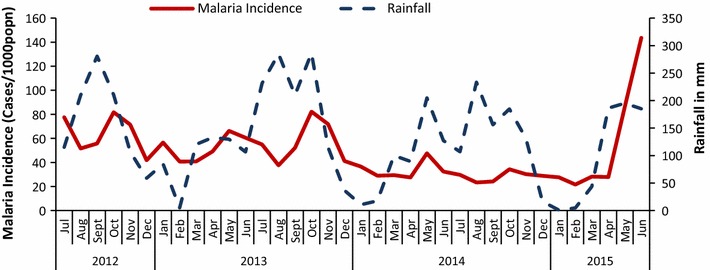
Malaria incidence among under-5 year-olds vis-à-vis rainfall in ten districts for period July 2012 to June 2015

Figure 4 shows malaria incidence among those under-5-years vis-à-vis rainfall in ten former IRS districts for period July 2012 to June 2015. In all the districts, there were two peak malaria transmission seasons per year (September to November and April to June), evidenced by a rapid increase in malaria incidence with peaks as high as 82 cases per 1000 population in October 2012/13 and 66 cases per 1000 population in May 2013, as shown in Figs. 2 and 3. This follows the two rainfall seasons, the first one being from February to May such as in 2015 where rainfall increased from as low as 5 mm in February to 195 mm in May and the second, July to October such as from 107 mm to 185 mm in 2014, that occur annually as shown in Fig. 4. Overtime, incidence reduced with peaks in 2014 considerably lower with 48 cases per 1000 population in May 2014 and 34 cases per 1000 population in October 2014 as shown in Figs. 2 and 3.

## Discussion

The findings in this study show a reduction in incidence of malaria among under-5 year-olds for up to 6 months following each implementation of IRS. However, in most cases IRS is shown to have been conducted during suboptimal periods and was not done until either the middle or end of the peak transmission period as shown in Figs. 1 and 2. According to the WHO operational manual on IRS, 2015, best practice is to schedule the completion of spray application to coincide with the buildup of vector populations just before the onset of the peak transmission season. The decrease in transmission might have thus been mainly due to natural transmission cycles rather than IRS. The poor timing of application of IRS could have in part been as a result of fluctuation in the usual dates of the rainy season leading to changes in malaria peak seasons.

Seven months after the withdrawal of IRS, there was an increase in incidence to levels above that of the last spray month. This reached epidemic proportions in June 2015 among the former IRS districts. This spike occurred after the peak in the rainfall season. The reduction in incidence of malaria for 6 months following IRS and its rise beyond this period re-emphasizes the known period of effectiveness of the carbamate used for IRS [3]. There seems to be an added effect on the reduction of incidence of malaria as a result of the mass distribution of LLINs as has been documented [3] where for the 2 months following the distribution, there was a further reduction in incidence.

Usage of LLINs at 53% among under-5-year-olds prior to the epidemic was not optimal for protecting the community against on-going transmission. There was not an adequate surveillance system in place to detect any increase or to trigger an appropriate outbreak response after the cessation of IRS. High net usage combined with a strong surveillance system, in addition to case management and intensified BCC has been highly recommended to safeguard gains made through IRS programmes. This could have helped prevent the epidemic.

Given the known residual effect of IRS that lasts 3–6 months, the resurgence would have been expected just after this period in 2014 in group II districts. However this occurred 14 months after the last IRS activity. In the group II districts, following the spray month, the IRR reduced for 4 months, after which it begun to increase 6 months post IRS. The incidence on the seventh month (November) post IRS surpassed that of the spray month as evidenced by the IRR, in line with the residual effectiveness of bendiocarb. This increase in IRR peaking in November 2014 followed two peaks in rainfall that occur in August and October as shown in Fig. 4. Following this peak in November, the IRR in group two districts started to reduce to a somewhat plateau in the next 2 months for the next 5 months with drops in the third (February) and fifth (April) month following the peak in November 2014. The drop in IRR after November 2014 followed a drop in rainfall from a peak of 185 mm in October 2014 to 17 mm in December hitting an all time low of 1 mm in January 2015. This low amount of rainfall is not suitable for breeding of mosquitoes and could have ultimately favored the drop in transmission hence IRR. Similarly, there was an increase in IRR from February to a peak in June which followed an increase in rainfall from January to a peak of 195 mm in May 2015.

In group I districts, following the spray month, the IRR reduced for 3 months, after which it begun to increase for the next 4 months after IRS, reaching a peak in the seventh month post IRS. This possibly shows effectiveness of IRS for a maximum of 6 months. Similar to group II districts, the drop in IRR 3 months post IRS follows a drop in rainfall from the spray month (November) to 3 months later in February 2015. Similarly, the increase 4 months following this decline, from February up to the peak in June 2015, was preceded by an increase in rainfall to a peak in May 2015.

The trend in the control districts was similar to that in the sprayed districts although there was a greater and steadier decline in the sprayed districts every month starting from the month following the last spray month up to the fourth month which could be attributed to the effect of IRS. It is hence plausible to say the decline in IRR for the sprayed districts was facilitated by both the end of transmission season and IRS.

The first peak in rainfall occurred in April 2015 just prior to the rise in cases culminating into an epidemic in June 2015. This peak in rainfall however, was lower than that of the previous 2 years. This indicates that the epidemic was not majorly due to the trend in rainfall. Since rainfall is one of the contributing factors for malaria transmission, this could have precipitated the rise in incidence to epidemic proportions in the presence of already weak control interventions post IRS.

This study had a number of limitations. The study assumed that malaria cases would be uniformly distributed within districts overtime and applied this assumption in adjusting the case numbers based on the reporting rates per district. There were gaps in cases reported for some of the earlier months. This was controlled by applying smoothing and interpolation methods to fill in missing figures between months prior to calculating the malaria incidence. The HMIS reporting system does not include patients attending all private clinics or malaria treated at home, so disease trends in health facilities does not reflect trends in the entire community. Incidence among under-5 year-olds was reported as trends based only on health facility reports in electronic HMIS for the period under review.

These findings have implications on policy for implementation of IRS. Prior to withdrawal of IRS activities, LLIN use should be optimal and surveillance of malaria at health facilities should be stepped up to closely monitor and safeguard gains made in malaria control. When carbamates are in use for IRS, spray activities should consistently be implemented every 6 months to achieve reduction in incidence of malaria. The completion of spray application should coincide with the buildup of vector populations just before the onset of the peak transmission season.

## Conclusions and recommendations

Prior to withdrawal of IRS, there was a sustained decline in incidence of malaria among under-5-year-olds for all the months between spray operations; however, the timing of spray activities was sub-optimal. Incidence reduced for 6 months post IRS, after which it increased until an epidemic was declared in June 2015 right after a peak in the rainfall season. This suggests a plausible link between the malaria epidemic, peak in rainfall and cessation of IRS. This study recommends that IRS should not be withdrawn especially where there is still considerable transmission and where there is sub-optimal uptake of malaria control interventions such as LLINs and malaria surveillance among other interventions. This study also recommends the schedule of completion of spray application to coincide with the buildup of vector populations just before the onset of the peak transmission season. The findings in this study cannot be generalized to all previously high endemic areas implementing IRS.

## Abbreviations

BCC: behavioural change communication
DHIS2: District Health Information Software2
eHMIS: electronic Health Management Information Systems
IPT: intermittent preventive treatment
IRR: incidence rate ratio
IRS: indoor residual spraying
LLINs: long-lasting insecticidal nets
VHT: village health teams
WHO: World Health Organization

## References

[1] WHO. World Malaria Report 2015. Geneva: World Health Organization; 2015. http://apps.who.int. Accessed 10 May 2016.

[2] Ministry of Health. The Uganda malaria reduction strategic plan 2014–2020. Kampala; 2014. http://health.go.ug/content/uganda-malaria-reduction-strategic-plan-2014-2020. Accessed 15 Jan 2016.

[3] Yimer F, Animut A, Erko B, Mamo H (2015). Past five-year trend, current prevalence and household knowledge, attitude and practice of malaria in Abeshge, south-central Ethiopia. MalarJ.

[4] WHO. Indoor residual spraying. An operational manual for indoor residual spraying (IRS) for malaria transmission control and elimination. 2nd Ed. Geneva: World Health Organization; 2015. http://www.who.int/malaria/publications/atoz/9789241508940/en/. Accessed 10 Feb 2016.

[5] WHO. World malaria report 2012. Geneva: World Health Organization; 2012. http://www.who.int/malaria/publications/world_malaria_report_2012/wmr2012_full_report.pdf. Accessed 14 July 2016.

[6] WHO. Guidelines for treatment of malaria. Geneva: World Health Organization; 2010. http://apps.who.int/medicinedocs/documents/s19105en/s19105en.pdf. Accessed July 2016.

[7] WHO. World Malaria Report 2010. Geneva: World Health Organization; 2010. http://www.who.int/malaria/world_malaria_report_2010/worldmalariareport2010.pdf. Accessed 12 Aug 2016.

[8] Ossè RA, Aikpon R, Gbédjissi GL, Gnanguenon V, Sèzonlin M, Govoétchan R (2013). A shift from indoor residual spraying (IRS) with bendiocarb to long-lasting insecticidal (mosquito) nets (LLINs) associated with changes in malaria transmission indicators in pyrethroid resistance areas in Benin. Parasit Vectors.

[9] Ministry of Health. Mid-Term Review of the 2010–2015 Malaria Strategic Plan. Kampala; 2014. https://www.k4health.org/sites/default/files/mtr_report_final.doc.Accessed 15 Jan 2016.

[10] Galactionova K, Tediosi F, Savigny D, Smith T, Tanner M (2015). Effective coverage and systems effectiveness for malaria case management in sub-Saharan African countries. PLoS ONE.

[11] Hamusse SD, Balcha TT, Belachew T (2012). The impact of indoor residual spraying on malaria incidence in East Shoa zone, Ethiopia. Glob Health Action.

[12] Curtis CF, Maxwell CA, Magesa SM, Rwegoshora RT, Wilkes TJ (2006). Insecticide-treated bed-nets for malaria mosquito control. J Am Mosq Control Assoc.

[13] President’s Malaria Initiative. Uganda Malaria Operational Plan FY 2015. 2014. https://www.pmi.gov/docs/default-source/default-document-library/malaria-operational-plans/fy-15/fy-2015-uganda-malaria-operational-plan.pdf?sfvrsn=3. Accessed 28 Nov 2016.

[14] Ministry of Health. Malaria Indicator Survey 2014–2015. 2015. Kampala, Uganda. https://dhsprogram.com/pubs/pdf/MIS21/MIS21.pdf. Accessed July 20 2016.

[15] WHO (2010). Effects of malaria control interventions on malaria.

[16] Uganda Bureau of Statistics. National Population and Housing Census 2014. Main Report, Kampala; 2016. http://www.ubos.org/onlinefiles/uploads/ubos/NPHC/NPHC%202014%20FINAL%20RESULTS%20REPORT.pdf. Accessed 30 July 2016.

[17] Kiberu VM, Matovu JKB, Makumbi F, Kyozira C, Mukooyo E, Wanyenze RK (2014). Strengthening district-based health reporting through the district health management information software system: the Ugandan experience. BMC Med Inform Decis Mak.

